# A prospective cohort study of the association between key family and individual factors and obesity status among youth

**DOI:** 10.1038/s41598-022-19585-8

**Published:** 2022-09-19

**Authors:** Liang Wang, Diana Morelen, Arsham Alamian

**Affiliations:** 1 Department of Public Health, Robbins College of Health and Human Sciences, Baylor University, Waco, TX 76798 USA; 2 Department of Psychology, East Tennessee State University, Johnson City, TN 37614 USA; 3 School of Nursing and Health Studies, University of Miami, Coral Gables, FL 33146 USA

**Keywords:** Medical research, Risk factors

## Abstract

There remains a significant gap in our knowledge of the synergistic nature of family dynamics, child characteristics, and child-rearing features in the etiology of obesity from childhood through adolescence. We assessed the associations of family dynamics (poverty, family structure), child characteristics (child temperament), and child-rearing features (maternal depression, maternal sensitivity, and type of child care) with the development of childhood obesity. Children (n = 1240) whose weights and heights were measured at least once for ten time points (from 2 years through 15 years) from the NICHD Study of Early Child Care and Youth Development were included. Generalized estimating equation (GEE) was used to examine the associations of family and individual factors with the childhood obesity after adjusting for covariates. Adjusted GEE models showed that living below poverty level was associated with an increased odds of obesity (odds ratio = 1.62, 95% confidence interval 1.05, 2.53). Among these key family and individual factors, poverty status was observed to be the strongest predictor of obesity of offspring across time. Findings highlight the importance of systemic-level public health changes in obesity reduction efforts and suggest that poverty-reduction based prevention and intervention are likely more effective targets than more individual/family specific targets.

## Introduction

The prevalence of obesity has reached a pandemic level in the world^1–3^. Obesity is defined as a disease by the World Health Organization (WHO) and is associated with the risk of morbidity from type 2 diabetes, hypertension, coronary heart disease, stroke, gallbladder disease, osteoarthritis, sleep apnea and respiratory problems, some cancers, impaired quality of life, psychosocial disturbance, and limited access to quality care^4,5^. Obesity among children and youth in the United States (U.S.) is a serious public health problem. While the importance of family contexts and parental factors in the development of childhood obesity has been recognized, there remains a significant gap in our knowledge of the synergistic nature of family dynamics, child characteristics, and child-rearing features in the etiology of obesity from childhood through adolescence. A thorough understanding of how multiple levels of risk intersect and accumulate over the course of the child’s life is key for effective prevention and intervention strategies.

The regulation of body weight and obesity is an exceedingly complex process that involves genetic, endocrine/regulatory, biological, behavioral, psychosocial, environmental, and economic factors^6,7^. Previous studies have focused largely on individual contributors such as dietary habits and activity level^8,9^, while some recent studies have focused on selected family characteristics such as socioeconomic status, single parent status, and parental body mass index (BMI)^10,11^. For instance, single parent households have been associated with a reliance on eating pre-prepared or fast-food meals and less time available for spending with children eating or engaging in outdoor physical activities^12^. Other studies have shown that children who lived with a single mother who entered a new union had healthier BMI trajectories than children whose mothers stayed single or become single; this finding emphasizes the importance of two-parent family contexts for children to develop and maintain a healthy weight status^13^. Similarly, a study found that poverty (defined as income-to-needs ratio < 200%) in the first 2 years of life was associated with increased risk of obesity by age 15 years^14^. However, the observed associations between family socioeconomic status and obesity have not been consistent across different studies^15–20^. Furthermore, the current literature on family level contributors to obesity is limited by cross-sectional designs, non-representative samples, and/or consideration of only a few risk factors rather than broader consideration of the interplay of complex environmental risk factors. It is crucial for future research to consider the interplay of individual and family level factors and the importance of developmental timing when considering risk.

The present study seeks to fill this gap through the use of a longitudinal national sample to examine the associations of family (poverty, family structure, maternal depression, maternal sensitivity, and non-maternal childcare) and individual (child temperament) level characteristics with the development of childhood obesity from 2 years through 15 years.

## Methods

### Study participants

This study utilized data from the National Institute of Child Health and Human Development’s Study of Early Child Care and Youth Development (NICHD SECCYD). The NICHD SECCYD is a comprehensive study of children including the contexts of their development. Participants in the NICHD SECCYD were recruited from hospitals at 10 research sites that were located in 10 different states in the U.S. Enrollment in the study involved three steps: (1) a hospital screening of mother-newborn dyads within 48 h after birth; (2) a 2-week phone call to mothers found to be eligible at screening; and (3) a 1-month interview with families that remained eligible after the 2-week phone call, agreed to the 1-month interview, and kept the interview appointment. Recruitment took place during the first 11 months of 1991. A total of 8986 mother-newborn dyads were screened in the hospital. Of the screened families, 3570 met exclusion criteria (mother under 18 years old; mother does not speak English; multiple births; family moving in less than 1 year; medical complication of baby or mother; family living too far away; refused 2-week phone call, etc.) and 5416 were eligible for the 2-week phone call. Among 3015 who were contacted by the 2-week phone call attempt, 1526 mothers were eligible for and agreed to an interview when the child was 1 month old. The reasons for not agreeing to the interview included child in the hospital [7 days (2.0%), moving within 3 years (3.0%), 3 unsuccessful calls (17.0%), refusal (21.3%), and other (6.1%)]. A total of 1364 of mothers completed the interview and were enrolled in the study^21^.

**Figure 1:**
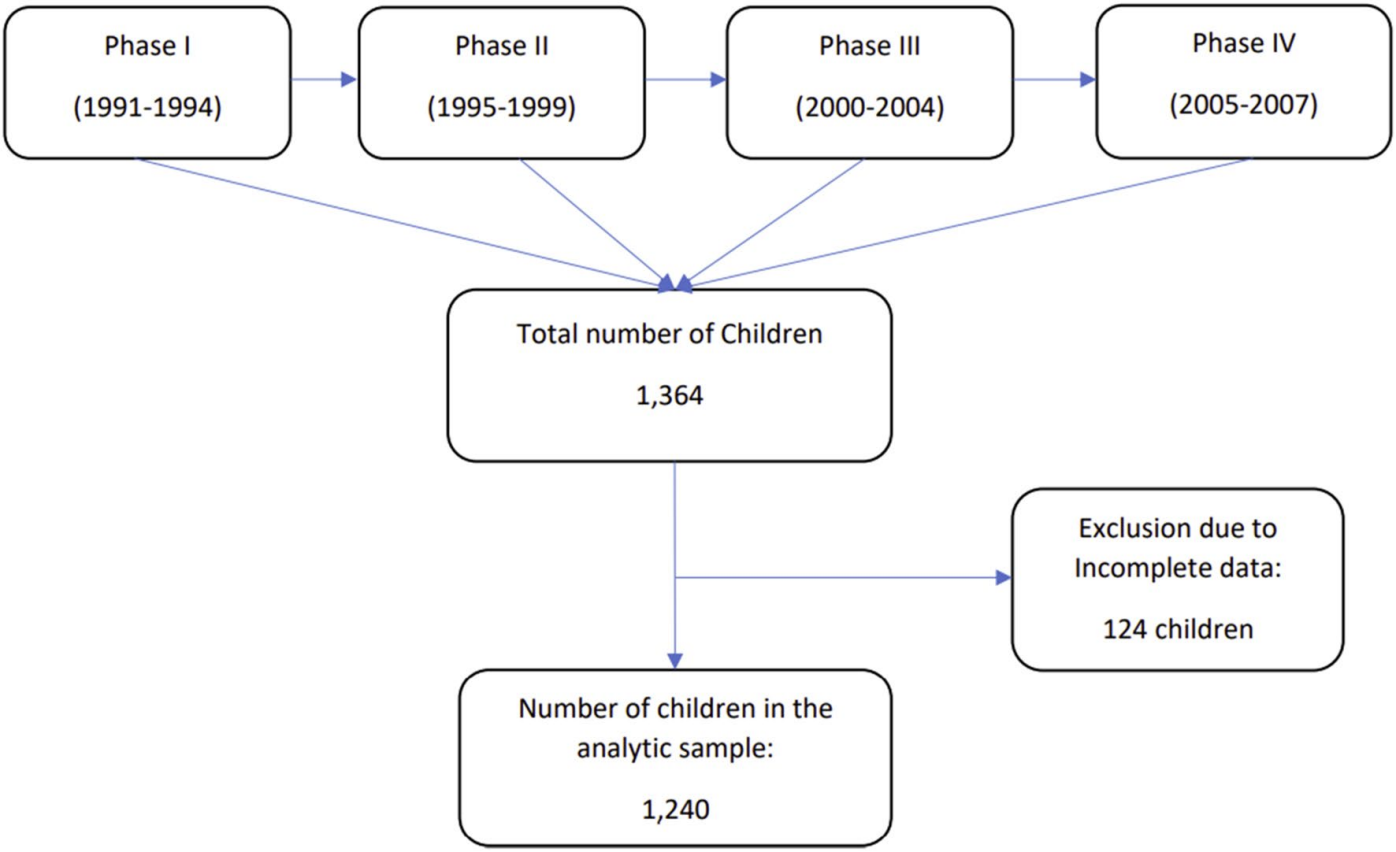
Study enrollment in NICHD SECCYD study.

The longitudinal design of the NICHD SECCYD followed a cohort of children at four continuous phases (1991–1994, 1995–1999, 2000–2004, and 2005–2007), from birth through age 15 years. Phase I (1991–1994) of the study followed 1364 children from birth to 3 years of age. Phase II (1995–1999) followed the 1226 children continuing to participate from 3 years of age to 2nd grade in school. Phase III (2000–2004) followed the 1100 children continuing to participate from 2nd to 6th grade. Phase IV followed 1009 children participated in the study through 9th grade (2005–2007). Additional information is available at https://www.icpsr.umich.edu/icpsrweb/ICPSR/series/00233^21^. The sample for this study was restricted to the children whose weights and heights were examined at one or more time points during the period from 2 years of age to 9th grade (15 years old) (Fig. 1). This study was approved by the Institutional Review Board of Baylor University and all methods were performed in accordance with the relevant guidelines and regulations. Informed consent for the original NICHD SECCYD was obtained from parents and assent was obtained from children when they were old enough to do so.

### Study variables

#### Obese status of adolescents

The outcome variable was childhood obesity, which is defined as a child whose BMI ≥ 95th percentile according to the U.S. Centers for Disease Control and Prevention (CDC)’s sex- and-age specific growth chart for the year 2000^22^. The BMI of children was calculated using measured height and weight obtained using standard protocols and procedures to measure height and weight during the interviews by the NICHD SECCYD staff^23^. Height was measured with children standing without shoes, feet together and their backs against a calibrated 7-foot measuring stick. Weight was measured using a physician’s two-beam scale. Scales were calibrated monthly using certified calibration weights. Weight was measured with children in minimal clothing and recorded twice, each time to the nearest 0.25 pound (0.1 kg).

#### Key family/individual factors

##### Poverty level

Family income level was measured as the ratio of income to needs, calculated as the total family income divided by the poverty threshold for their family size. Poverty was defined in this study by a median ratio of income to needs below 200%^24^; and it was dichotomized as at or above poverty line, or below poverty line.

##### Family structure

Marital status was defined at each assessment point as (a) mother single, not married; (b) mother separated or divorced; and (c) mother currently married; it was dichotomized as living together (c), or not living together (a and b).

##### Maternal depression

Maternal depressive symptoms were assessed longitudinally with the Center for Epidemiological Studies Depression Scale (CES–D). The CES-D is a 20-item, self-report depression scale developed to identify depression in the general population. A cutoff score of 16 or above is used as indicative of clinically significant depression^25^, which was used to dichotomize maternal depression (yes, no).

##### Maternal sensitivity

Maternal sensitivity was assessed by observing maternal behaviors during mother–child semi-structured interactions, when children were 6 months of age. Videotapes of the parent–child interaction from the 10 data collection sites were coded at a site uninvolved in data collection and by coders blind to family circumstances. Maternal behavior was rated on a series of 4- or 7-point rating scales, which were composited to create a summary measure of sensitive parenting, reflecting positive, nonintrusive, responsive, and supportive care. The composite was the sum of scores from scores on coded sensitivity to non-distress, intrusiveness (reverse scored), and positive regard. Internal consistency (Cronbach’s alpha) for the maternal sensitivity composite was above 0.70. For the purpose of this study, mothers were grouped into one of the two categories (sensitive or insensitive) using the median value of the sum score as a cut-off.

##### Child’s temperament

Child’s temperament was assessed by maternal report at 6 months using adapted versions of the Infant Temperament Questionnaire^26^. Items were designed to capture the temperament dimensions of approach, activity, intensity, mood, and adaptability. A total score was calculated for each subject by summing across all items. Adjustments were made for the number of items responded to in the questionnaire, to provide an overall unidimensional index consistent with the Thomas and Chess “difficultness” construct. An overall summary of ‘difficultness’ was calculated. The Cronbach’s alpha at 6 months was 0.81. Children were then grouped into three temperamental categories based on where they fell along the difficultness dimension (i.e., easy, average, and difficult) using the sample mean ± one standard deviation as cutoff values. This same approach was used in a recent analysis of the SECCYD temperament data^27^. Maternal ratings of temperament have been shown to be moderately stable across infancy and childhood^28^.

##### Child care experience

Type of care was examined. The primary care of each child was classified at each time point of assessment as: (1a) center care; (1b) home care (i.e., care in someone else’s home by a non-relative or relative other than the child’s grandparents); or (1c) parent care (i.e., care in the child’s home, including care by father). Quality of care was also examined. Group size and adult–child ratio of the care setting were used as a proxy for quality of care. However, the sample size for variables “group size of child care in center” and “adult–child ratio of child care in center” was 107 and 106, respectively, and thus these variables (for quality of care) were not used in the adjusted analyses.

### Covariates

Child’s sex, race (white, nonwhite), and birth weight in kg were treated as covariates in the adjusted analyses.

### Statistical analysis

By using the Chi-square and student’s t tests, characteristics of participants included in the analytic sample (n = 1240) were compared with participants not included because of incomplete data (n = 124). Simple descriptive statistics, including means, standard deviations, and proportions were used to describe body weight at different ages of children. The proportions of obese children were assessed in ten measures of BMI. Simple logistic regression was used to estimate an unadjusted odds of childhood obesity at the different assessment time points. To account for the within-subject correlation of response on dependent variables of different distributions^29^, we used the generalized estimating equation (GEE) method to estimate the parameters, because in comparison with traditional regression analysis at one time point (cross-sectional) or analysis of variance (ANOVA) to compare a certain outcome variable between two different time points, longitudinal GEE is an extension of the logistic regression model for correlated responses and takes into account that childhood obesity is repeatedly examined in the course of the study considering all repeated measurements of the outcome accounting for their dependency^30^. In this study, models were built using a GEE framework that is applicable to the binary response variable under study (obesity status); and the GEE model was used to account for within-subject correlation across the ten age-related time points of measurements for childhood obesity status.

In total, three models were used. In model 1, each key family/individual factor was included in the model to estimate its unadjusted associations with the odds of obesity from 2 years through age 15. Model 2 adjusted for all key family/individual factors. Model 3 adjusted for all key family/individual factors and further adjusted for covariates (i.e., child’s sex, ethnicity, and birth weight in kg). The stepped analyses provided some assessment of confounding and a better understanding of the associations of interest. GEE compares the same subjects at the ten time points, allowing for some missing data. Auto-regressive with first order was used as the working correlation structure in the analysis. All data analyses were performed using SAS version 9.4 statistical software (SAS Institute Inc., Cary, NC, USA).

## Results

Table 1 shows the characteristics of the study sample. The majority of families were at or above poverty line (87.34%), lived together (86.17%), and did not have maternal depression (83.55%). About half of mothers were sensitive to their child; home-based (41.20%) and parent-based (40.70%) were common types of child care for the families. With respect to the offspring, half of them were males (51.05%), more often white (81.6%) and having difficult temperament (62.44%), and the mean birth weight was 3.50 kg. Compared with mothers in the analytic sample (n = 1240), mothers in the excluded sample due to incomplete data (n = 124) were more likely to have depression (p < 0.0001); and children were more likely to have difficult temperament (p = 0.0353) and to be white (p = 0.0005). There was no statistically significant difference between analytic and excluded samples for poverty level, family structure, maternal depression, type of care, as well as child's sex and birth weight.

**Table 1:** Comparison of participants’ characteristics in the analytic sample with those not included due to incomplete data (n = 1364).

	Analytic sample^a^ n = 1240 (%)	Not in analytic sample n = 124 (%)	P value
Overall [n (%)]			
**Key family/individual factors**			
Poverty level^b^			0.3791
At or above poverty line (%)	1033 (87.34)	11 (100.00)	
Below poverty line (%)	143 (12.16)	0 (0.00)	
Family structure^b^			1.000
Living together (%)	1022 (86.17)	10 (90.91)	
Not living together (%)	164 (13.83)	1 (9.09)	
Maternal depression^c^			< 0.0001
No (%)	1036 (83.55)	123 (99.19)	
Yes (%)	204 (16.45)	1 (0.81)	
Maternal sensitivity^b^			0.3052
Sensitive (%)	598 (51.24)	2 (40.00)	
Insensitive (%)	569 (48.76)	3 (60.00)	
Child’s temperament^c^			0.0353
Easy (%)	44 (3.65)	1 (1.54)	
Average (%)	409 (33.91)	32 (49.23)	
Difficult (%)	753 (62.44)	32 (49.23)	
Type of care^c^			0.3016
Parent (%)	488 (40.70)	10 (31.25)	
Center (%)	217 (18.10)	9 (28.13)	
Home-based (%)	494 (41.20)	13 (40.63)	
**Other factors**			
Gender^c^			0.1360
Male (%)	633 (51.05)	72 (58.06)	
Female (%)	607 (49.95)	52 (41.94)	
Child ethnicity^c^			0.0005
White (%)	1012 (81.61)	85 (68.55)	
Nonwhite (%)	228 (18.39)	39 (31.45)	
Birth weight in kg (SD)^d^	3.50 (0.51)	3.42 (0.51)	0.0963
Group size of child care in center (SD)^d,e^	9.93 (4.09)	7.67 (1.73)	0.0051
Adult–child ratio of child care in center (SD)^d,f^	3.17 (1.28)	2.93 (1.28)	0.6215

*SD* standard deviation.

^a^Subjected included in analytic sample based on one or more time points of the ten measures of BMI (n = 1240).

^b^Fisher’s Exact test was used for p value.

^c^Chi-square test was used for p value.

^d^T test was used for p value.

^e^Sample size was only 107, not used in the adjusted models.

^f^Sample size was only 106, not used in the adjusted models.

Table 2 describes the distribution of mean BMI percentile, mean zBMI, and proportions of overweight and obese children at the time of assessment of children from 2 years through age 15. Generally, the proportion of overweight and obese children and mean BMI percentile increased with increases in the level of school grade, suggesting a positive relationship between grade level and weight. However, the increase in the prevalence of overweight and obese children reached a plateau from grade 5 to grade 8 and had a decrease at age 15.

**Table 2:** Child body weight at different ages of children.

	N	% Obesity ^a^	% Overweight ^b^	Mean BMI percentile (SD)	Mean zBMI (SD)
24 months	991	55 (5.6)	155 (15.6)	55.0 (27.4)	0.16 (0.94)
36 months	1090	69 (6.3)	203 (18.6)	54.8 (28.6)	0.17 (1.06)
54 months	1031	96 (9.3)	255 (24.7)	60.9 (27.3)	0.38 (0.99)
Grade 1	991	116 (11.7)	251 (25.3)	62.8 (26.7)	0.46 (0.96)
Grade 3	938	157 (16.7)	293 (31.2)	64.7 (27.9)	0.54 (1.05)
Grade 5	929	181 (19.5)	315 (33.9)	64.0 (29.3)	0.55 (1.08)
Grade 6	917	170 (18.5)	312 (34.0)	64.1 (29.4)	0.54 (1.11)
Grade 7	801	150 (18.7)	273 (34.1)	64.1 (29.0)	0.54 (1.09)
Grade 8	741	126 (17.0)	243 (32.8)	64.6 (28.2)	0.54 (1.05)
Grade 9 (15 years)	844	131 (15.5)	262 (31.0)	65.8 (26.7)	0.57 (0.99)

*BMI* body mass index, *SD* standard deviation.

^a^Obesity was defined as a BMI-for-age above the 95th percentile of the Centers for Disease Control and Prevention sex-specific BMI-forage growth charts.

^b^Overweight was defined as a BMI-for-age above the 85th percentile of the Centers for Disease Control and Prevention sex-specific BMI-forage growth charts.

Table 3 shows the associations of family and individual factors separately and jointly with the odds of childhood obesity using GEE after adjusting for covariates. GEE results of the unadjusted model (model 1) for each key family/individual factor, separately, showed that being below poverty line (odds ratio (OR) 2.15, 95% confidence interval (CI) 1.53–3.01) and not living together (OR 1.78, 95% CI 1.27–2.51) were associated with increased odds of obesity from 2 years through 15 years. GEE results of the model 2 that adjusted for all key family/individual factors together showed that being below poverty line (OR 1.78, 95% CI 1.15–2.73) was associated with increased odds of obesity from 2 years through 15 years. Model 3 further adjusted for child’s ethnicity, sex, and birth weight in kg and showed that being below poverty line (OR 1.63, 95% CI 1.05–2.53) remained positive association with the odds of obesity from 2 years through age 15. Additionally, being nonwhite and having a higher birthweight were positively associated with an increased odds of obesity.

**Table 3:** Association between family and individual factors and childhood obesity using GEE.

Key family and individual factors	Model 1 OR (95% CI)	Model 2 OR (95% CI)	Model 3 OR (95% CI)
**Poverty level**			
At or above poverty line	1 (referent)	1 (referent)	1 (referent)
Below poverty line	2.15 (1.53, 3.01)***	1.78 (1.15, 2.73)**	1.63 (1.05, 2.53)*
**Family structure**			
Living together	1 (referent)	1 (referent)	1 (referent)
Not living together	1.78 (1.27, 2.51)***	1.44 (0.94, 2.22)	1.42 (0.91, 2.22)
**Maternal depression**			
No	1 (referent)	1 (referent)	1 (referent)
Yes	1.32 (0.97, 1.81)	1.00 (0.70, 1.42)	0.99 (0.69, 1.40)
**Maternal sensitivity**			
Sensitive	1 (referent)	1 (referent)	1 (referent)
Insensitive	1.30 (1.00, 1.70)	1.13 (0.85, 1.49)	1.13 (0.85, 1.49)
**Child’s temperament**			
Easy	1 (referent)	1 (referent)	1 (referent)
Average	1.02 (0.77, 1.35)	0.89 (0.67, 1.19)	0.88 (0.65, 1.19)
Difficult	1.15 (0.58, 2.31)	0.95 (0.46, 1.99)	0.95 (0.44, 2.05)
**Type of care**			
Parent	1 (referent)	1 (referent)	1 (referent)
Center	0.82 (0.55, 1.20)	0.83 (0.56, 1.24)	0.83 (0.56, 1.23)
Home-based	1.21 (0.91, 1.62)	1.16 (0.86, 1.58)	1.17 (0.86, 1.60)

Model 1: unadjusted model for each key family/individual factor, separately.

Model 2: unadjusted model for all key family/individual factors, together.

Model 3: model 2 and further adjusted for child’s sex, ethnicity, and birth weight in kg.

*GEE* generalized estimating equation, *OR* odds ratio, *CI* confidence interval.

^a^Maternal depression was defined as CES-D score of 16 or greater.

^b^Obesity was defined as a BMI-for-age above the 95th percentile of the Centers for Disease Control and Prevention sex-specific BMI for age growth charts.

^c^Analysis of the GEE parameter estimates was based on empirical standard error and the use of auto-regressive with first order working correlation (AR1) structure, with obesity as a dichotomized outcome variable.

## Discussions

This study comprehensively examined the associations of key family and individual factors with the odds of obesity from 2 years through age 15 based on a prospective cohort study of mother–child pairs in the U.S. The main findings include (1) there was an overall positive relationship between child’s grade level and weight, but the increase in the prevalence of overweight and obesity reached a plateau from grade 5 to grade 8 and had a decrease at age 15; (2) being below poverty line was associated with increased odds of obesity from 2 years through age 15; and (3) being nonwhite and having a higher birthweight were positively associated with an increased odds of obesity.

Children do not develop in isolation, and they develop within the context of relationships^31^. Moving inward along Bronfenbrenner’s Ecological Systems Theory (1986)^32^, it is important to consider the parenting environment and how it contributes to the development of obesity. Two parental factors that have been shown to have robust associations with a host of child outcomes are parental depression and parenting styles^33,34^, and a growing body of literature ties these parental factors to the development of childhood obesity^35–37^. Unsurprisingly, the relations between obesity and parenting factors are complex, nuanced, and the literature is mixed regarding how parents influence the development and maintenance of childhood obesity. However, we did not observe an association between parenting style and the odds of childhood obesity. The finding is consistent with one study based on a U.S. national cohort that maternal-infant relationship quality was not associated with increased risk of obesity^15^. One possible reason is that maternal sensitivity was used as proxy of parenting style in this study. It is better to include a more comprehensive characteristics, e.g., authoritative, authoritarian, permissive, and neglectful, which are thought to be stable over time^38^. According to Maccoby and Martin^39^, these categories are defined by two dimensions: (1) demands for maturity or self-control (control/discipline) and (2) sensitivity and emotional involvement (warmth). For example, authoritative parenting, characterized by high control/discipline and high warmth, has generally been associated with adaptive child outcomes; whereas neglectful parenting, marked by low control discipline and low warmth, has generally been associated with unfavorable child outcomes^40,41^. Authoritarian parenting (high control/discipline and low warmth) and permissive parenting (low control/discipline and high warmth) are generally associated with poorer outcome when compared to authoritative parenting styles; however, these patterns are varied and depend, in part, on cultural considerations^42–44^. A systematic review of research published on this topic prior to 2010 indicated that children raised in homes with authoritative parenting tended to eat more healthily, be more physically active, and have lower BMI scores compared to children raised in homes with authoritarian, permissive, and/or neglectful parenting^45^. Further, this review highlighted the wide range of findings across studies likely due, in part, to the wide range of ways that studies conceptualized parenting styles and that some of the findings were moderated by socioeconomic status, maternal depression, and/or child sex^46,47^. Put together, the literature suggests that parenting styles likely influence child obesity through their influence on parenting behaviors and that it is important to consider issues related to diversity when investigating the influence of parenting style on child weight-related outcomes. Future studies are warranted to examine the association with parenting style with more categories and dimensions in addition to maternal sensitivity.

Parenting is challenging enough when a caregiver feels physically and mentally healthy and becomes even more challenging in the context of physical or mental health difficulties. Depression is one of the most common forms of mental health challenges that caregivers might experience with estimates that, on average, 15–20% of all women will experience depression during pregnancy or postpartum^48,49^ and approximately 10% of fathers will experience depression in the perinatal period^50^, with that rate increasing 5 folds in the context of maternal depression. Even beyond the first year postpartum, depression is one of the most common forms of mental illness in adults with approximately 15% of adults experiencing depression at some point in their lifetimes^51^ and risk for depression increasing exponentially when you add sociodemographic risk such as poverty^52^. However, we did not observe an association between maternal depression and the odds of childhood obesity, which is consistent with a review that included nine prospective studies for examining the prospective association between maternal depression and childhood weight status. The review concludes that chronic depression, but not episodic depression, was associated with greater risk of child overweight. Further research is needed to determine whether maternal depression influences child weight outcomes in childhood and adolescence and to investigate elements of the family ecology that may moderate the effect of maternal depression on child’s weight^53^. Furthermore, both maternal and paternal depression predicted a greater likelihood of adolescent obesity (age 17) after controlling for parental obesity^54^; and that maternal depression predicted lower parenting quality; which in turn, predicted children’s sedentary behaviors, healthy food choices, and leisure activities, which predicted child BMI^55^. Together, these studies illustrate the need for additional research including fathers^56^, the importance of considering mechanisms through which parental depression may influence child obesity status, and the complex relationship between parental depression symptoms and child obesity status.

In this study, being below poverty line was associated with increased odds of obesity from 2 years through age 15 after controlling for covariates. Our finding is consistent with previous studies, though most past findings are based on cross-sectional studies, e.g., using U.S. National Health and Nutrition Examination Survey^57–61^, California Health Interview Survey^62^, or U.S. National Survey of Children’s Health^63^. The present study used a national longitudinal study to examine the relationship between poverty and obesity that has been repeatedly measured during childhood with weight history. The definition of poverty used in this study aligns with the work of poverty scholars who argue that if absolute poverty thresholds were updated for changes in needs and consumption standards over time, the poverty line would be closer to 200% of the official level^64–66^. The U.S. National Institutes of Health Strategic Task Force on Obesity has put a priority on better understanding of the complex association between poverty (e.g., measured by socioeconomic status) and obesity^67^. In the future, more longitudinal studies of childhood poverty and its consequent risk of obesity throughout childhood and adolescence are needed, with aims to (1) explore the critical periods in childhood and adolescence during which poverty may have a greater influence on the incident obesity and (2) identify mechanisms through which poverty influences obesity risk. These efforts will help contribute to developing effective policies to prevent the incidence of childhood obesity^68,69^.

This study has several limitations. First, although the findings resulted from a well-designed longitudinal cohort study, causality cannot be assumed between exposure factors (e.g., income in early childhood) and the development of obesity due to the observational study design. Second, the dietary intake of children was not assessed, but previous studies suggest that it is a predictor of childhood obesity^70^. Third, some environmental factors (e.g., television viewing and computer use) that may play an important role in developing obesity risk were not controlled in the adjusted analyses due to lack of data. In addition, parents’ BMI and small for gestational age were not included as covariables although previous studies suggest a strong association between them^71^. Therefore, residual confounding remains possible due to exclusion of some predictors that may partly explain the observed associations. Fourth, some selection bias may have occurred due to incomplete data caused by lost to follow-up and missing data. It would be inconvenient to use only complete observations for the analysis due to varied missing data at each time point. Fifth, poverty is defined as income to needs ratio less than 200% which might be unfamiliar in an international setting. However, the poverty defined as an income to needs ratio of 2.00 or less represents an income at 200% of the poverty line or less, because many families near poverty also experience food insecurity and other conditions associated with an increased risk of obesity^72,73^. The definition of poverty aligns with the work of researchers who study poverty arguing that the poverty line would be closer to 200% of the official level if absolute poverty thresholds were updated for changes in needs and consumption standards over time^64–66^. In our analytic sample (n = 1024), children were less likely to be nonwhite, which imply that we may underestimate the association between being nonwhite and the odds of obesity of offspring. However, no difference was observed in poverty level between analytic and excluded samples, thus not influencing our finding for the positive association between poverty level and the odds of obesity. Despite these limitations, the study used prospective cohort study of mother–child pairs in the U.S. to provide epidemiological insight into how implications for the weight of children and could help in finding interventions to prevent the obesity epidemics. In addition, our study differs from other related studies that examined obesity using cohort designs. For instance, in a study from British cohorts in 1946, 1958, and 1950, while the authors used occupational social-class as an indicator of socio-economic status (SES), they did not consider the family income in their study to determine the SES of the family^74^. Further, our study differs in time and setting, and defines poverty based on income to needs ratio among the U.S. population. Fahrenkamp et al.^75^ assessed family level factors using the same cohort data as our study; however, they only assessed maternal depression at child’s age 9. The authors did not consider other family factors, and time points as compared to our analysis. In short, while there are some longitudinal studies using the same cohort data as our study (e.g., Lane et al.^76^), previous reports did not use the data from all four phases or all points of data collection. Hence, our study adds to the construct complexity (e.g., measurement income to needs ratio) and modeling complexity (e.g., individual, parenting, and family level factors examined simultaneously across multiple time points) to the existing literature base.

## Conclusion

Among these key family and individual factors, poverty status was observed to be the strongest predictor of obesity of offspring across time. Findings highlight the importance of systemic-level public health changes in obesity reduction efforts and suggest that poverty-reduction based prevention and intervention are likely more effective targets than more individual/family specific targets.

## Data Availability

The data that support the findings of this study are available from Inter-university Consortium for Political and Social Research (ICPSR, a unit within the Institute for Social Research at the University of Michigan), but restrictions apply to the availability of these data, which were used under license for the current study, and so are not publicly available. Data are however available from the authors upon reasonable request and with permission of ICPSR.
